# Role of the inferior frontal gyrus and impulsivity in approach motivation: A low-frequency repetitive transcranial magnetic stimulation study

**DOI:** 10.3758/s13415-026-01428-y

**Published:** 2026-04-07

**Authors:** Austin T. Moran, Timothy McCoy, Hilmar Zech, Philip A. Gable

**Affiliations:** 1 Department of Psychological and Brain Sciences, University of Delaware, Newark, DE USA; 2 Department of Psychiatry and Psychotherapy, Technische Universität Dresden, Dresden, Germany; 3 Department of Child and Adolescent Psychiatry, Psychosomatics and Psychotherapy, Centre of Mental Health, University of Würzburg, Margarete-Höppel-Platz 1, 97080 Würzburg, Germany; 4 Present Address: Department of Psychiatry, University of Pennsylvania, Philadelphia, PA USA

**Keywords:** Approach motivation, Impulsivity, Repetitive transcranial magnetic stimulation, Approach-avoidance task, Inferior frontal gyrus

## Abstract

Human actions are motivated by drives to approach positive outcomes or avoid adverse outcomes. Neurophysiological studies have associated the right-lateralized inferior frontal gyrus (IFG) with the regulation of approach motivation and individual differences in impulsivity, particularly in relation to deficits in motivational regulation. These studies suggest that the right IFG may inhibit approach-motivational tendencies. The current study investigated the causal role of the right IFG in motivational regulation and whether individual differences in impulsivity interact with the IFG to predict motivational regulation. The current study (*N* = 33) employed low-frequency repetitive transcranial magnetic stimulation to transiently inhibit the functioning of the right or left IFG before a mobile approach-avoidance task. Results revealed that inhibition of the right, but not the left, IFG causes greater approach-motivated behavior in individuals high in impulsivity. These results suggest that the right IFG acts as an inhibitory mechanism that plays a causal role in approach motivation when a “motivational amplifier” is present, such as greater trait impulsivity. This study also suggests that the right IFG is especially important for regulating motivation in individuals with deficits in inhibitory control. These findings inform models of impulsivity and personality by revealing the causal role of the right IFG as a neural substrate of motivational inhibition and by demonstrating how individual differences in impulsivity can impact motivational regulation.

**Supplementary Information:**

The online version contains supplementary material available at 10.3758/s13415-026-01428-y.

## Introduction

Humans constantly encounter choices that elicit a range of emotions, motivations, and reactions driven by impulses to pursue positive outcomes or avoid negative outcomes. Approach behavior occurs in the presence of potential rewards, goal achievement, or stimuli that ensure safety and security. In contrast, avoidance behavior occurs in the presence of impending or experienced punishments and potential threats (Carver & Scheier, 2008; Gray, 1970, 1987; McNaughton & Gray, 2000; Lang et al., 1998). When these systems become activated, individuals integrate information regarding the value, likelihood, and magnitude of each potential reward or punishment (Champion, 1961; Elliot & Thrash, 2002; Kakoschke et al., 2015). During this process, motivation regulation plays a pivotal role in altering behavior and facilitating the intricate balance between different approach and avoidance motivations. Impulsivity, the inverse of motivational regulation, reflects deficits in motivational control. Together, these mechanisms influence how individuals adapt their behavior to better align with overarching goals and values (Aupperle et al., 2015; Quartz, 2009; Rolls & Grabenhorst, 2008).

### Neural substrates of approach-avoidance motivation

An abundance of research has investigated approach-avoidance behaviors, as well as decision-making by utilizing animal models (Friedman et al., 2015; Millan, 2003) and human neuroimaging (Aupperle et al., 2015; Bach et al., 2014; O’Neil et al., 2015; Park et al., 2011; Talmi et al., 2009). Early animal models observed approach-avoidance conflict by associating rewards (e.g., food) with punishment (e.g., mild shock) (Millan, 2003; Millan & Brocco, 2003). They demonstrated significant anatomical connections to motivational processing through lesioning of subcortical structures (e.g., the amygdala) and cortical regions (e.g., the prefrontal cortex) (Kopchia et al., 1992; Millan, 2003; Möller et al., 1997; Resstel et al., 2008; Yamashita et al., 1989). Similarly, human neuroimaging has provided more detailed accounts of cognitive processing in response to threat-related stimuli, reward processing, and decision-making, which have been inspired by these earlier animal models (Berkman & Lieberman, 2010; Prévost et al., 2011; Schlund et al., 2011; Spielberg et al., 2008).

Various regions of the prefrontal cortex (PFC) are linked to motivation, decision-making, and executive functions (Enticott et al., 2006; Logan et al., 1997). Past research utilizing electroencephalography (EEG) and functional magnetic resonance imaging (fMRI) demonstrated that the PFC hemispheres are asymmetrically related to motivational systems (Allen et al., 2004; Cook et al., 1998; Pizzagalli et al., 2005). Frontal asymmetry is a measure used to quantify the difference in activity between the left and right frontal lobes of the brain. It is predominantly measured by using EEG and is especially relevant to the study of emotion, motivation, and psychopathology (Harmon-Jones & Allen, 1997). Specifically, greater relative left frontal asymmetry is associated with approach motivation, while greater relative right frontal asymmetry is linked with avoidance motivation (Gable & Poole, 2014; Harmon-Jones, 2004; Sutton & Davidson, 1997). This asymmetry in hemispheric neural organization may underlie observed trait differences in impulsive tendencies and motivational drive.

An increasing body of literature suggests that impulsivity is an important risk factor for psychopathology (Brezo et al., 2006; Johnson et al., 2016). Impulsivity is a broad, multifaceted construct composed of sensation-seeking (the tendency to pursue novelty and excitement), response inhibition (difficulty suppressing automatic responses), lack of premeditation (acting without considering future consequences), lack of perseverance (struggling with sustained effort), and urgency (the tendency to act impulsively in response to emotion; Whiteside & Lynam, 2001; Whiteside et al., 2005; Cyders & Smith, 2007; Sharma et al., 2014). Multiple facets of trait impulsivity have been linked to greater relative left frontal asymmetry, including trait sensation-seeking (Santesso et al., 2008), response inhibition (Schiller et al., 2014), risk-taking (Gianotti et al., 2009), positive urgency (Gable et al., 2015), as well as negative urgency, lack of premeditation, and lack of perseverance (Neal & Gable, 2016). EEG source localization has suggested that heightened impulsive behavior linked to greater relative left frontal asymmetry resulted from reduced activity in the right inferior frontal gyrus (IFG; Gable et al., 2018; Neal & Gable, 2016). However, past work investigating asymmetry using EEG has not been able to determine causal associations between impulsivity and relative frontal asymmetry.

The inferior frontal gyrus is closely associated with the regulation of motivational and behavioral control. Lesion studies support the notion that the right inferior frontal gyrus (IFG) is critical for inhibitory control, specifically during prepotent response inhibition (Aron et al., 2003; Rieger et al., 2003). The right IFG appears to implement inhibitory control via a wider prefrontal-basal ganglia network, which includes subcortical projections to the subthalamic nucleus and substantia nigra, thereby inhibiting the thalamus (Aron & Poldrack, 2006). Although many studies have investigated the inhibitory function of the right IFG, its causal role in motivational regulation remains relatively unknown. Additionally, most previous research has overlooked the relationship between individual differences in impulsivity and the asymmetric activation of the IFG. Determining the mechanisms of inhibition related to motivation is crucial for understanding decision-making and executive control.

### Transcranial magnetic stimulation of the frontal cortex

Transcranial magnetic stimulation (TMS) is a noninvasive method of modulating cortical activity that employs electromagnetic induction to disrupt neural function transiently (Klomjai et al., 2015). Low-frequency TMS, or stimulation ≤ 1 Hz, has inhibitory effects on targeted neurons and has been utilized to observe the relationship between neural regions and motivation effectively (Pell et al., 2011; Robertson et al., 2003).

Previous TMS research has primarily investigated the roles of various PFC regions in motivation. Specifically, studies have examined the significance of the right dorsolateral prefrontal cortex (DLPFC) in risk-taking behavior (Knoch et al., 2006) and decision-making (Rolle et al., 2022), as well as functional specializations of cognitive control in prefrontal and premotor cortices (Chambers et al., 2007). These studies highlighted the importance of the right IFG on inhibition, specifically in the presence of heightened response competition caused by a prepotent motor response (Chambers et al., 2007). These results associate the right IFG with selective inhibitory control and suggest that the right IFG acts as an inhibitory mechanism that plays a causal role in approach motivation, particularly when there is a “motivational amplifier” such as trait impulsivity present. However, few studies utilizing TMS have evaluated the direct mechanisms underlying motivational systems specific to approach-avoidance regulation in relation to individual differences.

### Current experiment

In the current experiment, participants completed a mobile approach-avoidance task, where they were instructed to approach (pull) or avoid (push) pictures of approach-motivating desserts or neutral objects displayed on a handheld tablet (Zech et al., 2022). We applied low-frequency repetitive transcranial magnetic stimulation (rTMS) during two separate sessions to inhibit the function of the left or right IFG transiently. This allows for the examination of the IFG’s causal role in approach motivation and motivational regulation during an approach-avoidance task (AAT). Given the role of the right IFG in inhibition and avoidance behavior (Aron et al., 2014; Chambers et al., 2007; Swann et al., 2012), we hypothesized that low-frequency rTMS when applied to the right (vs. left) IFG would result in reduced regulation of approach motivation and greater approach-motivated behavior. Specifically, stimulation to the right, compared with the left IFG, should impair inhibition of motivation, resulting in faster reaction times during approach movements when reacting to positive stimuli.

Although previous work suggests a link between the IFG and individual differences in trait impulsivity, most research has neglected to examine the specific connection between individual differences and activation of neural regions associated with motivational systems. Behavior change may not result solely from stimulation, and individual differences in trait impulsivity may serve as an amplifier, which increases approach-motivated behavior. Individual differences in impulsivity were assessed by using the UPPS Impulsivity Questionnaire (Whiteside et al., 2005). We predicted that individual differences in impulsivity would interact with hemispheric stimulation to predict greater approach motivation. Specifically, individuals with greater impulsivity should exhibit greater approach motivation during right hemisphere inhibition compared to left hemisphere inhibition. This study uniquely bridges the gap between research focused on individual differences and existing literature on the neural substrates of motivation.

## Materials and methods

### Subjects

Forty-five (20 females) right-handed adult volunteers were recruited from the community. A total of nine participants were excluded from analysis owing to incorrect task completion (e.g., only completing one session, too many task errors, or equipment issues). Additionally, a robust fit Huber test identified outliers in the coil distance to the target in three participants’ sessions, which were excluded to ensure proper stimulation of the IFG. The final sample consisted of 33 participants. The average participant age was 23.59 years (standard deviation [SD] = 7.1).

Before participating in the study, each participant underwent screening for TMS contraindications using a standardized form (see Supplemental Material 1). Exclusion criteria comprised 1) a history of clinically diagnosed psychiatric disorders, such as mania, psychosis, or depression; 2) current medications that reduce seizure threshold (refer to Supplemental Material 2 for the full list); 3) neurological issues, including seizures, history of epilepsy in the participant or a first-degree relative, stroke, traumatic brain injury with more than 5 min of unconsciousness, or prior brain surgery; 4) metallic implants or medical devices; and 5) other contraindications, such as tinnitus, pregnancy, headaches, sleep deprivation, or recent alcohol intake (Rossi et al., 2009). Any participant reporting psychiatric diagnoses or medication use was excluded. However, subclinical psychiatric symptoms in otherwise healthy participants were not systematically assessed. Written informed consent approved by the Institutional Review Board Ethics Committee (#1,878,420–2) was obtained upon participants' arrival. Each participant was individually tested across two sessions and reimbursed $12.50 USD per hour for participation.

### UPPS-P behavioral impulsivity scale

Participants completed the UPPS-P Behavioral Impulsivity Scale (Cyders & Smith, 2007). The UPPS-P Behavioral Impulsivity Scale assesses trait impulsivity by evaluating two distinct areas of impulsivity: executive-control deficits and emotional rash action. Executive-control deficits include subscales: lack of premeditation and lack of perseverance, while emotional rash action includes sensation-seeking, negative urgency, and positive urgency (Cyders & Smith, 2008; Whiteside et al., 2005). The scale comprises 59 items, each rated on a 4-point Likert scale (1 = strongly disagree, 4 = strongly agree). Each subscale of the UPPS-P Behavioral Impulsivity Scale has been previously linked to different types of risky behavior (Coskunpinar et al., 2013; Fischer et al., 2008).

### Experimental design and procedure

The experiment consisted of two sessions separated by at least 24 h to account for possible interference from any prior stimulation, with an average time between sessions being around 8 days (M = 7.92). To minimize variability between sessions, both sessions were scheduled at similar times (within 1 to 2 h) to reduce variability in the time of day of stimulation. In addition, procedures were identical in both sessions—with the exception of stimulation hemisphere—to maintain consistency between time periods. Participants completed a TMS contraindication checklist at each start to confirm no changes in medication, substance use, or health factors affecting cortical excitability. The resting motor threshold (rMT) was remeasured before stimulation to account for fluctuations in stimulation sensitivity. Stimulation targets were remapped by using the Brainsight Neuronavigation System (Rogue Research), with coil position and angle continuously monitored to ensure consistency.

After completing the UPPS-P scale, participants were comfortably seated, and the experimenter determined the resting motor threshold (rMT) using the Rossini-Rothwell (R-R) relative-frequency estimation method (Rossini et al., 2015; Tranulis et al., 2006). Prior to the task, participants received one period of low-frequency rTMS to either the right or left IFG (Knoch et al., 2006). The current study did not include a sham condition because of the confound between the subjective experience between sham and real stimulation, which undermines its effectiveness as a blind control (Duecker & Sack, 2015). After stimulation, participants completed a mobile approach-avoidance task (AAT), followed by self-reports that assessed trait impulsivity and trait motivation. In their second session, participants underwent the same procedure and level of stimulation. However, the stimulation target was changed to the opposing hemisphere relative to the first session. The stimulation order was randomly assigned.

### Approach-avoidance task

After rTMS stimulation, participants used an Android tablet to complete the mobile AAT. This task consisted of a modified version of the AAT, initially developed by Solarz (1960) and revised by Zech et al. (2020), which was adapted to run on tablets and smartphones (Zech et al., 2022). Reliability of the mobile AAT is comparable to that of traditional versions of the approach-avoidance task, which use keyboards or joysticks (Zech et al., 2022). Specifically, the task shows moderate to high test–retest reliability (ICCk = 0.73) and adequate split-half reliability (r = 0.85–0.73). These reliability results were replicated within the current study (see *Results* sections for mobile AAT reliability). Participants were instructed to hold the handheld tablet approximately one foot from their chests and to keep it perpendicular to the floor. In addition to these instructions, the experimenter demonstrated the correct distance and completion of the push and pull movements, ensuring the participant completed the task correctly before beginning the practice trials. Practice trials provided additional demonstration and feedback for task completion.

During this task, participants were instructed to either pull the mobile tablet towards themselves or to push the tablet away when shown pictures of appetitive desserts (e.g., baking cookies, chocolates, cakes) or neutral objects (e.g., stapler, clock, rocks). After each picture was presented, participants were instructed to return the tablet to a neutral starting position by bringing it back to its original distance of approximately one foot from their chest. The experimenter provided affirming or corrective feedback after each block, as needed, to ensure continued proper execution. Importantly, the motor movements of pulling and pushing are associated with aspects of approach and avoidance motivation systems, which are amplified or conflicting depending on the stimuli being used (Zech et al., 2022). Participants completed two task blocks (e.g., congruent and incongruent), with the block order being randomly assigned. During congruent task blocks, participants were instructed to pull the tablet towards themselves when shown pictures of desserts and to push the tablet away when presented with object pictures. During incongruent task blocks, participants were instructed to pull the tablet towards themselves when shown object pictures and to push the mobile tablet away when shown dessert pictures. Each block included five dessert pictures and five object pictures, which were repeated four times. This yielded a total of 80 trials. A fixation cross preceded each stimulus and remained on the screen for 1.5 s (Zech et al., 2020). The mobile AAT detected reaction time and reaction force.

Reaction times were hypothesized to be the most sensitive behavioral measure of motivated behavior. Scores were inverted for analysis (1/RT), such that larger inverted reaction times corresponded to faster responses, while smaller inverted reaction times indicated a slower response. Scores were inverted to aid interpretability and facilitate comparison with previous studies that utilized the mobile AAT (McCoy et al., 2024; Zech et al., 2020, 2023).

### Approach-avoidance task reliability

In the current sample, trials were divided into odd and even within-participant conditions and adjusted using the Spearman-Brown formula for split-half reliability. Reliability was generally satisfactory to excellent across most conditions: Session 1/Session 2: Dessert Pull rSB = 0.82/0.84, Dessert Push rSB = 0.82/0.91, Object Pull rSB = 0.80/0.84, Object Push rSB = 0.60/0.90. Furthermore, internal consistency was evaluated for each condition with acceptable to strong reliability for session 1 (Dessert Pull α = 0.82, Dessert Push α = 0.87, Object Pull α = 0.82, Object Push α = 0.73), and for session 2 (Dessert Pull α = 0.85, Dessert Push α = 0.86, Object Pull α = 0.84, Object Push α = 0.85). Split-half reliability for the main outcome, the approach bias difference score (Dessert Pull—Object Pull) across both sessions, was excellent (rSB = 0.86). This high split-half reliability suggests that the approach bias difference score has strong internal consistency in this sample.

### Repetitive transcranial magnetic stimulation

During each session, participants received a single 15-min 1 Hz rTMS train of 900 pulses, administered to either the left or right IFG (counterbalanced by session). Low-frequency rTMS was used to suppress the excitability of targeted cortical regions for several minutes after the completion of the rTMS train (Robertson et al., 2003). Prior to stimulation, each participant's rMT was determined to control for inter-participant variability in sensitivity to TMS. Stimulation intensity was set at 92% of the individuals’ rMT with stimulation intensities averaging 55% (SD = 5.23) of maximal stimulator output. Stimulation was performed using a Magistim Rapid^2^ Stimulator (The Magstim Company Ltd., Whitland, UK) and a 70-mm figure-of-eight Air-cooled Coil (D702 Coil). The coil was manually positioned via individualized scalp registration and target site localization using Montreal Neurological Institute (MNI) Coordinates. The coil was fixed tangentially to the scalp and oriented at 60° relative to the central sulcus. A Magstim AFC Support Stand (model/PN 4735–00; The Magstim Company Ltd.) was utilized to ensure the coil placement remained in the proper location throughout the stimulation period. All rTMS parameters were within the recommended safety guidelines (Wassermann, 1998).

### Anatomical localization

The targeted cortical regions included the right and left IFG. The left IFG was defined as (x =  − 39, y = 45, z = 7) and the right IFG was defined as (x = 42, y = 45, z =  − 6). These coordinates were taken from previous neuroimaging studies that localized specific brain structures (i.e., IFG, right dlPFC) associated with approach-avoidance conflict (Zorowitz et al., 2019). Participants’ heads were mapped using known anatomical landmarks for positioning of stimulation location using the Brainsight Neuronavigation System (Rogue Research) and a Northern Digital Polaris Vicra camera. A three-dimensional record of anatomical landmarks and scalp topography for each participant was registered and then warped to an MNI ICBM 152 Average Brain. Individual target site localization was then achieved by applying the MNI coordinates for the left and right IFG for each individual. Coil positioning was also confirmed throughout stimulation using optical tracking via the Brainsight Neuronavigation System (Rogue Research) in conjunction with the Northern Digital Polaris Vicra camera. The average target distance error of the coil was 0.95 mm (SD = 0.25 mm). In addition, the mean angular and twist errors were 4.15° (SD = 1.55°) and 10.69° (SD = 11.00°), respectively. These stimulation errors are within those commonly reported in TMS research (Herwig et al., 2001).

### Planned analysis

A 2 × 2 repeated measures ANOVA was performed to assess whether dessert pictures were more positive, approach-motivating, and elicited greater craving than object pictures shown during the task. Following this, a repeated-measures 2 × 2 × 2 ANOVA (Picture Type × Movement × Hemisphere Stimulation) was conducted for Accuracy, Force, and Reaction Time. Lastly, owing to reaction time being the variable most associated with motivation in the approach-avoidance tasks, we performed linear regressions for approach movements (pull) reaction time toward dessert images vs. pulling object images to determine whether those higher in impulsive traits, specifically executive-control deficits (lack of premeditation & lack of perseverance) as opposed to emotional rash action (sensation-seeking, positive urgency, & negative urgency) moderated the pull movements toward dessert pictures after inhibiting the right vs. the left IFG. To address the issue of multiple comparisons across the five UPPS-P impulsivity subscales, we applied Bonferroni corrections separately within two theoretically defined families: 1) cognitive impulsivity/executive-control deficits (Lack of Premeditation, Lack of Perseverance; corrected *α* = 0.05/2 = 0.025), and 2) emotional impulsivity/emotional rash action (Positive Urgency, Negative Urgency, Sensation Seeking; corrected *α* = 0.05/3 = 0.0167). This family-wise approach was justified because cognitive and emotional impulsivity psychometrically map onto separate dimensions and may involve different neural mechanisms (Cyders & Smith, 2008; Whiteside & Lynam, 2001).

## Results

### Preliminary analysis

Nine participants were excluded from the analysis due to improper task completion or equipment malfunction. In addition, a robust fit Huber test identified outliers for coil distance to target in three participants’ sessions, which were excluded to ensure proper stimulation of the IFG. The final sample comprised data from a total of 33 participants, who were included and analyzed using TIBCO Statistica and R with an alpha level set at 0.05. Post hoc sensitivity analyses were conducted using G*Power to estimate the minimum detectable effect sizes for the current sample (*N* = 33). For the repeated-measures 2 × 2 × 2 ANOVA (Picture Type × Movement × Hemisphere), the design aimed to detect within-subject interaction effects of at least partial *η*^*2*^ = 0.11 (*f* = 0.35), corresponding to a medium effect size at 80% power (*α* = 0.05).

Because underpowered studies are a growing concern in personality neuroscience research (DeYoung et al., 2022), we also investigated the power of our sample to detect moderation effects of individual differences. For the linear regression models testing impulsivity moderation effects, the sensitivity analysis indicated sufficient power to detect interaction effects with *f*^*2*^ values of 0.158 or larger, reflecting medium effect sizes. Additionally, paired-samples *t*-tests (e.g., comparing right vs. left IFG conditions) were powered to detect Cohen’s *d* = 0.51 or greater. These estimates indicate that the study was sufficiently powered to detect medium-to-large effects; however, smaller effects may have remained undetected.

### Picture ratings

A repeated measures ANOVA was conducted to determine whether dessert pictures were more positive, approach-motivating, and evoked more craving than object pictures shown during the task. Participants reported that dessert pictures were more positive (M = 2.37, SD = 1.18) than object pictures (M = 1.46, SD =.08), *F*(1, 31) = 21.235, *p* < 0.001, *η*^*2*^ = 0.406. Dessert pictures were rated as more approach-motivating (M = 4.69, SD = 1.13) than object pictures (M = 2.17, SD = 1.03), *F*(1, 31) = 118.319, *p* < 0.001, *η*^*2*^ = 0.792. Participants also reported that dessert pictures elicited higher levels of craving (M = 5.47, SD = 0.87) than object pictures (M = 4.29, SD = 1.03), *F*(1, 31) = 34.18, *p* < 0.001, *η*^*2*^ = 0.524. A dependent samples t-test revealed no significant difference in picture ratings between sessions (*t* values < 1.991, *p* values > 0.055).

### Reaction time results

**Fig. 1:**
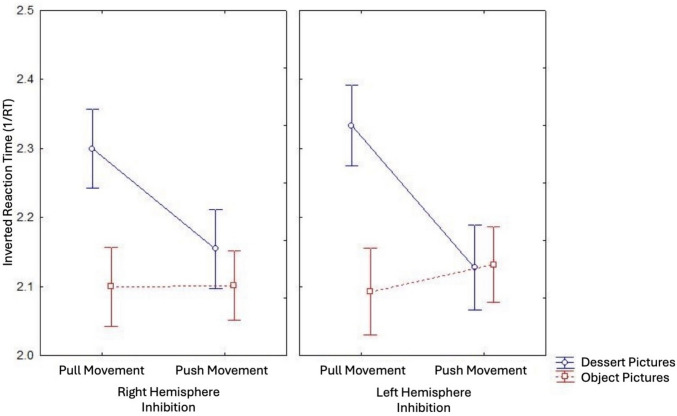
Repeated ANOVA of inverted reaction time. *Note.* A two (picture type) by two (movement) effect on participants’ inverted reaction time. Error bars represent standard error

A 2 (Picture Type) by 2 (Movement) by 2 (Hemisphere: left IFG vs. right IFG) repeated measures ANOVA revealed a main effect of picture type (dessert vs. object), with participants responding faster to dessert pictures than object pictures, *F*(1, 31) = 126.455, *p* < 0.001, *η*^*2*^ = 0.803 (Fig. 1). A main effect of movement (push vs. pull) was also found, suggesting that participants were faster to pull the tablet than to push it away, *F*(1, 31) = 13.004, *p* < 0.001, *η*^*2*^ = 0.296. However, there was no significant main effect of hemisphere stimulation, *F*(1, 31) = 0.055, *p* = 0.816, *η*^*2*^ = 0.002, or any two-way interactions involving hemisphere stimulation, *p’s* > 0.119, *η*^*2*^ < 0.028.

There was a significant two-way interaction between picture type and movement type for reaction time, *F*(1, 31) = 8.458, *p* = 0.007, *η*^*2*^ = 0.214. Post-hoc analyses revealed that participants were significantly faster at pulling dessert pictures towards themselves as opposed to pushing them away, *t* (31) = 4.118, *p* = 0.001, and were faster to pull dessert pictures than to pull object pictures, *t* (31) = 5.960, *p* < 0.001.

The three-way interaction (Picture Type x Movement x Hemisphere) was nonsignificant, *F*(1, 31) = 0.893, *p* = 0.352, *η*^*2*^ = 0.028. These results suggest participants were more approach-motivated and less avoidance-motivated to dessert pictures than object pictures.

### Force results

A 2 (Picture Type) by 2 (Movement) by 2 (Hemisphere: left IFG vs. right IFG) repeated-measures ANOVA was performed on movement force. A main effect of movement was observed, *F*(1, 31) = 4.22, *p* = 0.049, *η*^*2*^ = 0.12, indicating that participants exerted significantly more force when pulling than when pushing either image type. The main effect of picture type was marginal, *F*(1, 31) = 3.97, *p* = 0.055, *η*^*2*^ = 0.114, indicating a trend toward greater force when pulling dessert images. There was no significant main effect of hemisphere stimulation, *F*(1, 31) = 0.45,* p* = 0.509, *η*^*2*^ = 0.014, and none of the two-way interactions reached significance (*p* values > 0.42, *η*^*2*^ <.013.

The three-way interaction among picture type, movement, and hemisphere stimulation was also non-significant, *F*(1, 31) < 0.01, *p* = 0.985, *η*^*2*^ < 0.001. These results suggest that, overall, pulling movements generated greater force than pushing movements.

### Accuracy results

A 2 (Picture Type) by 2 (Movement) by 2 (Hemisphere: left IFG vs. right IFG) repeated-measures ANOVA was conducted on accuracy. There was a significant main effect of movement, *F*(1, 31) = 6.09, *p* = 0.019, *η*^*2*^ = 0.16, indicating that participants were more accurate during pull trials compared to push trials. The main effects of picture type, *F*(1, 31) = 2.46, *p* = 0.127, *η*^*2*^ = 0.071, and hemisphere stimulation, *F*(1, 31) = 1.74, *p* = 0.197, *η*^*2*^ = 0.051, were not statistically significant.

No significant two-way interactions were found (*p* values > 0.101). The three-way interaction involving image type, movement, and hemisphere stimulation was also not significant, *F*(1, 31) = 2.85, *p* = 0.101, *η*^*2*^ = 0.082. These results suggest that pull movements were more accurate than push movements.

To assess the potential influence of stimulation order, we conducted an additional repeated measures ANOVA of accuracy, including stimulation order (right-first vs. left-first) as a between-subjects factor. No main effects or interactions involving stimulation order were significant (all *p* values > 0.3), indicating that the order of stimulation did not influence accuracy.

Reaction time was most sensitive to the type of stimulus and movement related to motivation. Because reaction time was a more sensitive behavioral measure, subsequent analyses focused on reaction time as the primary dependent measure.

### Effects of individual differences in impulsivity on IFG inhibition

Generalized linear models were constructed to test for interactions between personality variables and lateralized inhibition of the IFG. To test whether individual differences in impulsivity influenced the effect of hemispheric inhibition on reaction time, we calculated difference scores by subtracting the left-hemisphere (dessert pull − object pull) from the right-hemisphere (dessert pull − object pull) to aid in interpretability. This reflects the relative increase in pull reaction times toward dessert following right IFG inhibition, with higher scores indicating a stronger bias toward dessert cues when the right hemisphere was stimulated. Importantly, the current sample demonstrated strong internal consistency and test–retest reliability, indicating the acceptability of using this difference score in the current sample. When combining data from both stimulation sessions, the difference score (dessert pull – object pull) showed excellent internal consistency (rSB = 0.86), indicating that participants’ relative approach bias toward dessert images was stable across trials compared to objects.

**Fig. 2:**
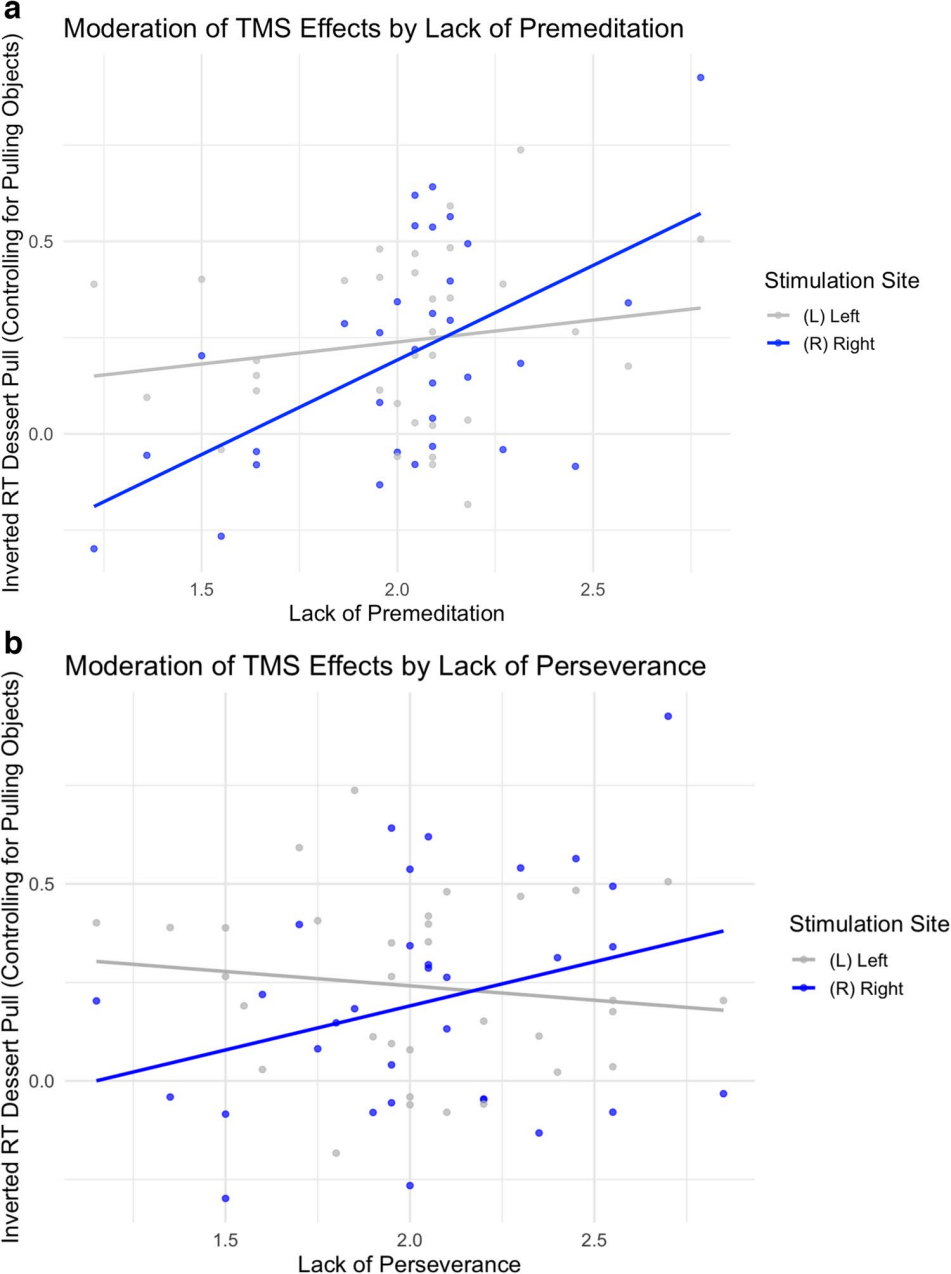
**a** Interaction between lack of premeditation and hemisphere in predicting approach behavior. *Note.* The relationship of reaction time to pull dessert pictures, controlled for pulling object pictures to individual differences in lack of premeditation after right (blue) or left (grey) hemispheric inhibition. **b** The interaction between lack of perseverance and hemisphere in predicting approach behavior. *Note.* The relationship of reaction time to pull dessert pictures, controlled for pulling object pictures to individual differences in lack of perseverance after right (blue) or left (grey) hemispheric inhibition

Reaction times for pulling dessert pictures showed a significant interaction between inhibited hemispheres and a lack of premeditation, *F*(1, 31) = 6.094, *p* = 0.019, *R*^*2*^ = 0.164 (Fig. 2a). Therefore, individuals high in lack of premeditation were more likely to have faster reaction times when pulling dessert pictures compared to left IFG inhibition. This suggests that right hemispheric inhibition resulted in greater approach motivation to dessert pictures when individuals were high in lack of premeditation. These findings imply that hemispheric inhibition caused greater approach-motivated behavior only when individuals were high in lack of premeditation. Reaction times for pushing dessert pictures showed no significant interaction between hemispheric inhibition and lack of premeditation, controlling for reaction times to pushing objects *F*(1, 31) = 0.321, *p* = 0.575), suggesting that the influence of lack of premeditation was unique to approach movements toward dessert pictures.

Reaction times for pulling dessert pictures also had a significant interaction between inhibited hemispheres and lack of perseverance, controlling for reaction times to pulling objects, *F*(1, 31) = 5.829, *p* = 0.022, *R*^*2*^ = 0.158 (Fig. 2b). After right IFG inhibition, individuals who were high in lack of perseverance exhibited faster reaction times when pulling dessert pictures compared with after left IFG inhibition, suggesting that right hemispheric inhibition resulted in greater approach motivation to dessert pictures among individuals high in lack of perseverance. This suggests that hemispheric inhibition led to greater approach-motivated behavior in individuals who lack perseverance. Reaction times for pushing dessert pictures showed no significant interaction between the inhibited hemisphere and lack of perseverance, controlling for reaction times to pushing objects, *F*(1,31) = 0.334, *p* = 0.568, suggesting that the influence of lack of perseverance was unique to approach movements toward dessert pictures.

Reaction times for pulling dessert pictures showed no significant interaction between inhibited hemispheres and other individual difference variables, including positive urgency* F*(1, 31) = 3.625, *p* = 0.066), negative urgency *F*(1, 31) = 3.366, *p* = 0.076), or sensation-seeking *F*(1, 31) = 2.056, *p* = 0.162). While effects with positive urgency and negative urgency were marginal, they did not reach the level of statistical significance. In addition, reaction times for pushing dessert pictures, when controlling for pushing objects, had no significant interaction between inhibited hemispheres and any of these facets of impulsivity (*F* values < 0.44 and *p* values > 0.425). No comparable interactions emerged when analyzing object stimuli, suggesting that the effects of impulsivity on approach motivation were specific to motivationally salient pictures of desserts. Both cognitive impulsivity effects remained significant after correction (Lack of Premeditation: *p* = 0.019 < 0.025; Lack of Perseverance: *p* = 0.022 < 0.025), while all emotional impulsivity subscales remained nonsignificant (all *p* values > 0.066).

Finally, to determine whether these same executive facets of impulsivity moderated how participants responded to appetitive stimuli after right vs. left IFG inhibition, the same moderation analysis was performed with task accuracy as the dependent variable. No impulsivity facets moderated the relationship between hemisphere and accuracy (*p* values > 0.31). These results indicate that impulsivity specifically influenced approach-related reaction times rather than task accuracy.

## Discussion

Research on frontal asymmetry has suggested that the left and right IFG, as well as individual differences in impulsivity, may play a crucial role in motivated behavior. This current study sought to utilize TMS to investigate the function of the IFG in selective inhibition causally, as well as the role individual differences play in enhancing or inhibiting motivation. Low-frequency TMS to the IFG, administered before conducting a tablet-based AAT, revealed that decreased right IFG activity increased approach motivation towards dessert pictures in individuals with elevated impulsivity. Specifically, participants who scored higher on the UPPS-P subscales, lack of premeditation and lack of perseverance, were quicker to pull dessert pictures after receiving inhibitory low-frequency stimulation to the right IFG as opposed to the left IFG.

Because impulsivity is related to deficits in inhibition, effortful control, and executive functions, impulsivity can be an inverse measure of an individual’s motivational control system (Enticott et al., 2006; Logan et al., 1997). These results suggest that an inhibited right IFG, combined with decreased motivational control, led to enhanced approach behavior. Together, these findings inform current models of motivational behavior, suggesting that the right IFG may have a causal role in approach motivation when a “motivational amplifier,” such as impulsivity, is present.

Individual differences in impulsivity may influence the relationship between neural correlates of approach behavior and motivation regulation (Gable et al., 2015). Various neuroimaging studies have shown that the right IFG contributes to motivational control systems (Coan & Allen, 2003; Gable & Harmon-Jones, 2008; Harmon-Jones, 2006; Harmon-Jones & Allen, 1997; Harmon-Jones et al., 2010; Sutton & Davidson, 1997), which govern aspects of decision-making by inhibiting approach motivation (Kelley & Schmeichel, 2016). However, previous research largely neglects specific connections between individual differences and the activation of neuronal regions associated with motivational systems. Assessing facets of impulsivity concerning their neural correlates can teach us about psychological disorders related to impulsivity, including substance use disorders, ADHD (Costa Dias et al., 2013), schizophrenia (Ouzir, 2013), and OCD (Summerfeldt et al., 2004), among others.

The current results reveal a unique interplay between executive dimensions of impulsivity (lack of premeditation and lack of perseverance) and the right IFG when approaching appetitive dessert images within the current task design. Lack of premeditation refers to the tendency to act impulsively without regard to consequences or potential risks, while lack of perseverance refers to one’s ability to maintain attention during goal-directed behavior. Identifying that differences in individual facets of impulsivity can impact approach-motivated movements when the right IFG is inhibited, enhances our understanding of how different facets of impulsivity contribute to personality, motivation, and behavior.

The results of the current study suggest that individual differences in facets of impulsivity have an impact on motivational control governed by the right IFG. More specifically, levels of lack of premeditation and lack of perseverance are influential to an individual’s approach behavior and motivation regulation when the right IFG activity is decreased. These interactions imply that the right IFG primarily inhibits impulsive behavior. Individuals with higher levels of impulsivity may be less inhibited and more prone to approach-motivated behavior due to lower activation of the right IFG. Our results also suggest that frontal asymmetry in the region of the IFG relates to individual impulsivity traits, because these interactions were significant only after right IFG inhibition, and not left IFG inhibition. This is supported by previous research, which localized the relationship between specific facets of impulsivity (e.g., positive urgency) and frontal asymmetry to diminished activity in the right IFG (Gable et al., 2015). In addition, positive urgency, lack of premeditation, and lack of perseverance have also been localized to the right cingulate gyrus (Neal & Gable, 2016), which plays a significant role in error detection (O’Connell et al., 2007; Taylor et al., 2006; Van Veen et al., 2001) and has functional connections to the IFG (Du et al., 2020). The subdimensions of Lack of premeditation and lack of perseverance reflect aspects of impulsivity related to specific forms of conscientiousness (Gullo et al., 2014), which may clarify why an interaction was only found for these dimensions of impulsivity.

These findings support models that explain impulsivity as a behavior that happens when our natural desire to act is stronger than our ability to control ourselves. This suggests that facets of impulsivity may be linked to the brain's ability to regulate or inhibit innate and automatic responses. More broadly, this supports the notion that the ability to regulate motivational drives, may depend on areas of the right frontal cortex to engage. By connecting brain areas related to inhibition with real-time behaviors influenced by impulsivity, this study advances the integration of psychological theories about traits with scientifically based models of behavior. Along with others investigating personality neuroscience, we see the investigation of neural correlates of personality traits as an exciting area for investigation (DeYoung et al., 2022).

While this study has many strengths, it also has limitations within its current design. Although the left IFG served as a direct comparison to right IFG stimulation, this does not allow us to isolate the behavioral effects using a sham control condition. A sham control condition could have helped to determine whether the observed effects stemmed specifically from cortical inhibition or general procedural effects. However, the current study did not include a sham condition, because previous researchers have reported that participants can often distinguish between sham and real stimulation, which undermines its effectiveness as a blind control (Duecker & Sack, 2015).

Also, while our approach bias difference score demonstrated excellent split-half reliability (rSB = 0.86), the broader AAT literature has documented variable test–retest reliability for approach-avoidance metrics; some studies report low ICCs for the mobile AAT (Zech et al., 2022). Notably, Zech et al. (2022) used the mobile AAT monthly for 7 months in uncontrolled conditions outside the laboratory, whereas the current study included only two sessions, separated by approximately a week, under different experimental manipulations (left vs. right IFG stimulation). Importantly, measurement variability may contribute to inconsistent findings across AAT studies and represents an important area for methodological development.

Another noteworthy limitation is the accuracy of stimulation targeting. Although we utilized Neuronavigation, anatomical landmarks, and personalized scalp registration to minimize localization errors, stimulation sites mapped to MNI coordinates may not fully account for individual differences in head size, cortical folding, or sex. This is especially relevant given the small size of the IFG and its proximity to regions, such as the dorsolateral prefrontal cortex. Despite a low average error of less than 1 mm, some spread to adjacent prefrontal regions (e.g., dlPFC) is possible.

Additionally, the absence of a baseline measure limits our ability to observe the net change in behavior after stimulation. However, the current study was designed to mitigate concerns about participant fatigue and behavioral alterations following repeated exposure to the motivational stimuli. Future research would benefit from including a baseline to evaluate the magnitude and direction of changes in participants’ behavior following stimulation. Lastly, the sample size may limit the generalizability of the findings, specifically regarding the personality-based moderation effects. This limitation is not uncommon in personality neuroscience research, highlighting the need for future replication in larger samples.

Overall, the current study found that the right IFG acts as an inhibitory mechanism, and individual differences in impulsivity can serve as an inverse measure of motivational control. An inhibited right IFG combined with decreased motivational control was found to enhance approach behavior. This work revealed that individual differences are influential on approach-motivated behavior for individuals with deficits in inhibitory control. In addition, the right but not the left IFG appears to be causally related to inhibitory control. These findings help to inform models of impulsivity by highlighting the role of the right IFG as a neural substrate of motivational inhibitory control. Most importantly, individual differences in facets of impulsivity appear to exacerbate a lack of inhibitory control. One’s internal impulses and ability to react to external stimuli may not only be strongly influenced by situational context but also by differences in neural activity and impulsive tendencies. By learning more about individual differences and their neural correlates, we may be able to develop novel models of personality and construct more individualized treatments for pathologies associated with aspects of motivational control.

## Supplementary Information

Below is the link to the electronic supplementary material.

Supplementary file1 (PDF 251 kb)

## Data Availability

All data and materials are available upon request from the 1 st author.

## Code availability

The code used to administer the task will be made available upon request from the 1 st author.
