# Supplementary material for: Role of the inferior frontal gyrus and impulsivity in approach motivation: A low-frequency repetitive transcranial magnetic stimulation study
Source: Cogn Affect Behav Neurosci. 2026 Apr 7;26(4):1478–91. doi: 10.3758/s13415-026-01428-y (PMC13385091; doi:10.3758/s13415-026-01428-y)
Supplement: Supplementary file 1 — Supplementary file1 (PDF 251 kb) [file 13415_2026_1428_MOESM1_ESM.pdf]

Supplemental Material 1

Inclusion and Exclusion Criteria Checklist

Inclusion criteria: (*Must answer* **yes** *to all questions to be eligible for participation*)

18 years of age or older?	Yes	No
Able to speak and read English?	Yes	No
Right-Handed?	Yes	No
Have Normal/Corrected Normal Eyesight?	Yes	No

Exclusion criteria: (*Must answer* **no** *to all questions to be eligible for participation*)

History of or active neurological problems, including seizures?	Yes	No
History of psychiatric disorders (clinically assessed), including mania, psychosis or depression?	Yes	No
History of seizures or of epilepsy in first degree relative?	Yes	No
History of head injury with unconsciousness lasting more than 5 minutes?	Yes	No
History of stroke?	Yes	No
Previous brain surgery?	Yes	No
Other medical or neurologic conditions in which a seizure would be particularly harmful (e.g., increased intracranial pressure)	Yes	No
Metallic hardware such as cardiac pacemakers, cochlear implants, implantable medical pumps, ventriculo-peritoneal shunts, deep brain stimulators, or intracardiac lines.	Yes	No
History of tinnitus (persistent ringing or buzzing in the ears)?	Yes	No
Consumption of any drugs and/or medicines listed in the Drug Contradiction Document, or that are known to lower the seizure threshold?	Yes	No
Ever had an adverse reaction to TMS?	Yes	No
Currently suffer from frequent or severe headaches?	Yes	No
Participated in any other TMS or tDCS sessions today?	Yes	No
Consumed one or more alcoholic beverages within the last hour?	Yes	No
Are you currently sleep deprived (less than 4 hours of sleep)?	Yes	No

For female subjects:

Are you pregnant or experiencing a late menstrual period?	Yes	No

For Review Only

Supplemental Material 2

TMS Drug Contraindication List

Note that most major brand names are listed in parentheses. However, if they are not sure, please look up the brand name and ensure that the scientific name of the drug is not on the list below.

Alcohol (within one hour of testing)

Amitriptyline (Elavil)

Amphetamines (Adderall, Dexedrine, Focalin, Methylin, Ritalin, Vyvanse)

Chlorpromazine (Thorazine, Largactil, etc.)

Clozapine (Clozaril, etc.)

Cocaine

Doxepin (Deptran, Sinequan)

Foscarnet (Foscavir)

Gamma-hydroxybutyrate (GHB)

Ganciclovir (Cytovene)

Imipramine (Tofranil)

Ketamine

Maprotiline (Ludiomil)

MDMA (ecstasy)

Nortriptyline (Aventil, Pamelor)

Phencyclidine (PCP, angel's dust),

Ritonavir (Norvir)

Theophylline (Theolair, Slo-Bid)

Any other medication prescribed for antidepressant medication or medication prescribed to decrease seizure thresholds

Participant has reviewed the list and stated they are not currently using any of the listed medications.

Subject ID: ______________________

Researcher ID: ___________________

Testing Date: ____________________

For Review Only

Inclusion and Exclusion Criteria Checklist

Transcranial Magnetic Stimulation

Subject ID: ____________________________

Researcher ID: __________________________

Subject Age: ___________________________

Testing Date: __________________________

Inclusion criteria: (*Must answer* **yes** *to all questions to be eligible for participation*)

Is the subject:

18 years of age or older?	Yes	No
Able to speak and read English?	Yes	No
Right-Handed?	Yes	No
Have Normal/Corrected Normal Eyesight?	Yes	No
Has the subject been informed that they cannot receive TMS if they are pregnant?	Yes	No

Exclusion criteria: (*Must answer* **no** *to all questions to be eligible for participation*)

Does the subject have any of the following:

History of or active neurological problems, including seizures?	Yes	No
History of psychiatric disorders (clinically assessed), including mania, psychosis or depression?	Yes	No
History of seizures or of epilepsy in first degree relative?	Yes	No
History of head injury with unconsciousness lasting more than 5 minutes?	Yes	No
History of stroke?	Yes	No
Previous brain surgery?	Yes	No
Other medical or neurologic conditions in which a seizure would be particularly harmful (e.g., increased intracranial pressure)	Yes	No
Metallic hardware such as cardiac pacemakers, cochlear implants, implantable medical pumps, ventriculo-peritoneal shunts, deep brain stimulators, or intracardiac lines.	Yes	No

For Review Only

History of tinnitus (persistent ringing or buzzing in the ears)?	Yes	No
Consumption of any drugs and/or medicines listed in the Drug Contradiction Document, or that are known to lower the seizure threshold?	Yes	No
Ever had an adverse reaction to TMS?	Yes	No
Currently suffer from frequent or severe headaches?	Yes	No
Participated in any other TMS or tDCS sessions today?	Yes	No
Consumed one or more alcoholic beverages within the last hour?	Yes	No
Are you currently sleep deprived (less than 4 hours of sleep)?	Yes	No

For female subjects:

Are you pregnant or experiencing a late menstrual period?	Yes	No

For Review Only

Form reviewed by Experimenter:

Print name	Signature	Date

TMS Drug Contraindication List

Note that most major brand names are listed in parentheses. However, if they are not sure, please look up the brand name and ensure that the scientific name of the drug is not on the list below.

Alcohol (within one hour of testing)

Amitriptyline (Elavil)

Amphetamines (Adderall, Dexedrine, Focalin, Methylin, Ritalin, Vyvanse)

Chlorpromazine (Thorazine, Largactil, etc.)

Clozapine (Clozaril, etc.)

Cocaine

Doxepin (Deptran, Sinequan)

Foscarnet (Foscavir)

Gamma-hydroxybutyrate (GHB)

Ganciclovir (Cytovene)

Imipramine (Tofranil)

Ketamine

Maprotiline (Ludiomil)

MDMA (ecstasy)

Nortriptyline (Aventil, Pamelor)

Phencyclidine (PCP, angel's dust),

Ritonavir (Norvir)

Theophylline (Theolair, Slo-Bid)

Any other medication prescribed for antidepressant medication or medication prescribed to decrease seizure thresholds

Participant has reviewed the list and stated they are not currently using any of the listed medications.

Subject ID: ____________________

Researcher ID: _________________

Testing Date: __________________

For Review Only
